# Fast Thermocycling in Custom Microfluidic Cartridge for Rapid Single-Molecule Droplet PCR

**DOI:** 10.3390/s23249884

**Published:** 2023-12-17

**Authors:** Hirokazu Takahara, Hayato Tanaka, Masahiko Hashimoto

**Affiliations:** Department of Chemical Engineering and Materials Science, Faculty of Science and Engineering, Doshisha University, 1-3 Tataramiyakodani, Kyotanabe 610-0321, Kyoto, Japan

**Keywords:** droplet microfluidics, droplet PCR, polydimethylsiloxane (PDMS), single-molecule PCR

## Abstract

The microfluidic droplet polymerase chain reaction (PCR), which enables simultaneous DNA amplification in numerous droplets, has led to the discovery of various applications that were previously deemed unattainable. Decades ago, it was demonstrated that the temperature holding periods at the denaturation and annealing stages in thermal cycles for PCR amplification could be essentially eliminated if a rapid change of temperature for an entire PCR mixture was achieved. Microfluidic devices facilitating the application of such fast thermocycling protocols have significantly reduced the time required for PCR. However, in microfluidic droplet PCR, ensuring successful amplification from single molecules within droplets has limited studies on accelerating assays through fast thermocycling. Our developed microfluidic cartridge, distinguished for its convenience in executing single-molecule droplet PCR with common laboratory equipment, features droplets positioned on a thin glass slide. We hypothesized that applying fast thermocycling to this cartridge would achieve single-molecule droplet PCR amplification. Indeed, the application of this fast protocol demonstrated successful amplification in just 22 min for 30 cycles (40 s/cycle). This breakthrough is noteworthy for its potential to expedite microfluidic droplet PCR assays, ensuring efficient single-molecule amplification within a remarkably short timeframe.

## 1. Introduction

For the quantification of nucleic acid molecules, the microfluidic droplet polymerase chain reaction (PCR) method conducts PCR within water-in-oil (W/O) droplets created through the utilization of microfluidic technology. After PCR amplification, these droplets are evaluated for fluorescence, distinguishing between fluorescence-positive and fluorescence-negative ones. The proportion of positive droplets to the total analyzed droplets is then determined. Based on this proportion, the copy number of nucleic acid molecules in the sample solution is calculated using Poisson regression. It is noteworthy that, unlike conventional quantitative real-time PCR, microfluidic droplet PCR allows the quantification of absolute amounts of nucleic acid molecules without relying on standard calibration curves. Moreover, microfluidic droplet PCR excels in quantification accuracy and precision compared to real-time PCR, especially in the low-copy-number range [1,2,3,4,5,6,7]. This method proves groundbreaking for enabling precise measurements of nucleic acid molecules, providing both high sensitivity and accuracy in analysis. Because of its capacity for massively parallel singleplex amplification of target sequences, even from single copies, microfluidic droplet PCR has unlocked previously unattainable applications, such as absolute quantification of nucleic acid molecules [6,8,9,10,11,12,13], detection of extremely infrequent mutations [14,15,16,17], cost-effective aptamer selection [18,19], and enrichment of specific loci for large-scale targeted sequencing [20].

In microfluidic droplet PCR, a large number of W/O droplets prepared by using a microfluidic chip are thermocycled on-chip [21,22,23] or off-chip (i.e., conventional heat block/microcentrifuge tube cycling) [24,25,26,27] for target sequence amplification within the droplets. Regardless of the format employed, it has been common to spend hours repeating temperature cycles with traditional protocols that conventionally employ temperature holding for 30–60 s at each of the set temperatures for denaturation, annealing, and extension in a thermocycle. As demonstrated approximately three decades ago [28], however, PCR can be processed much more rapidly with essentially no holding time at denaturation and annealing temperatures—if immediate temperature equilibration of the PCR reaction mixture at each of the targeted temperatures can be achieved through using small sample volumes, thin sample container walls, and high surface area-to-volume ratios of the sample exposed to the container wall. Fast PCR cycling was first reported in a system using thin glass capillary tubes as reactor vessels because of their low thermal capacity, which enables rapid thermal equilibrium to be reached through heat transfer [28]. Since this original research was conducted, many microfluidic devices for performing rapid PCR with limited sample volume have been reported (some of these studies are summarized in recent review articles [29,30]). Nevertheless, few studies have been conducted to expedite microfluidic droplet PCR through rapid PCR cycling. The limited research on this topic includes recent studies by Jalilli et al. [31] and Yin et al. [32], who were successful in achieving fast thermocycling through the use of sophisticated thermocycling systems that they developed independently. Although the achieved thermocycling in these studies is faster than what was accomplished in our current study, we believe that the evaluation of the developed experimental systems should consider various perspectives such as cost, ease of use, versatility, and flexibility, not just the speed of thermocycling. We will delve into this discussion in more detail in a later section, comparing the results obtained.

The workflow of droplet PCR involves sequential processing of the following three discrete steps: (1) droplet generation, (2) PCR amplification, and (3) measurement of the fluorescence intensity of the thermocycled droplets. Transfer of droplets between these steps usually requires careful and time-consuming manual droplet handling with micropipettes, as has been the case in our previous studies [25,33,34]. We recently developed a new microfluidic cartridge for droplet PCR that eliminates the need for manual transport of droplets between the major steps of droplet PCR analysis [35], enabling facile execution of single-molecule droplet PCR. The new cartridge features four layers with a thin glass slide (0.15 mm thick) as the bottommost layer. We delivered the droplets generated in the polydimethylsiloxane (PDMS) microfluidic layer, serving as the top layer, onto the glass slide in an online fashion. After a large number of droplets accumulated on the glass slide, we placed the cartridge directly on a flatbed heat block of a thermocycler to enable PCR amplification. The cartridge could then be transferred to the stage of a conventional fluorescence microscope for imaging of thermocycled droplets on glass slides. We applied a conventional temperature protocol with tens of seconds of hold time at denaturation and combined anneal/extension temperatures to the cartridge to ensure amplification of the target sequence. We confirmed sufficient heat transfer from the heat block to the droplets through the glass slide by PCR amplification within the droplets, even from single template molecules. The objective, hypothesis, results, and conclusions of this study, conducted against the above background, are outlined below.

Objective: Among the key steps in the droplet PCR process mentioned above, the PCR amplification step stands out as the most time-consuming. Therefore, by shortening the amplification step of PCR, it becomes feasible to expedite droplet PCR analysis. Classical studies have demonstrated that maintaining the PCR mixture at denaturation and annealing temperatures for a certain duration (normally tens of seconds) is unnecessary if the temperature of the entire PCR mixture is rapidly brought to these temperatures. The aim of this study is to accelerate droplet PCR analysis by implementing such a rapid thermocycling protocol in our microfluidic cartridge.

Hypothesis: Considering the structural features of our microfluidic cartridge, wherein droplets generated on PDMS microfluidic sheets are positioned on a thin glass surface in contact with the heat block of the thermal cycler, it is expected that the droplet temperature will quickly respond to the temperature changes of the heat block. Consequently, even when applying the previously mentioned rapid temperature cycling protocol, successful PCR amplification from single molecules within the droplets (critical for achieving absolute quantification based on Poisson regression in droplet PCR) can be anticipated.

Results: In our previous study, we applied a standard cycling protocol with hold times of 30 s and 60 s at denaturation and annealing temperatures, respectively. This protocol required 67 min for 30 thermal cycles. In contrast, in the present study, we demonstrated the feasibility of performing droplet-based PCR amplification from single molecules even when applying a fast cycling protocol, which omitted hold times at denaturation and annealing temperatures. The fast cycling protocol allowed us to achieve PCR amplification within a mere 22 min (40 s/cycle). Although the efficiency of PCR amplification slightly decreased when compared to the standard cycling protocol, clear discrimination between the fluorescence-positive droplet population and the fluorescence-negative droplet population was possible. This enabled precise quantification of the target molecules based on Poisson regression.

Conclusions: Reducing the size of the droplets used in this study is expected to lead to an increased surface area-to-volume ratio, facilitating a more rapid adjustment of droplet temperature. Our microfluidic cartridge stands out for its capability to facilely perform droplet PCR using commercially available thermal cyclers. The smaller size of the droplets within our cartridge implies that their temperature can closely track the increasing temperature change rates of upcoming commercial thermal cyclers, which have been progressively accelerating over the years. Consequently, without requiring fundamental modifications to the basic structure of this microfluidic cartridge, it is anticipated that an even faster droplet PCR can be achieved.

## 2. Materials and Methods

### 2.1. PCR Reagents

For PCR amplification, Lambda DNA (New England Biolabs Japan, Tokyo, Japan) served as the template. A defined copy number of the template was introduced into a 25 µL solution comprising 1× Colorless GoTaq Flexi Buffer (Promega K.K., Tokyo, Japan), 0.5 g/L bovine serum albumin (Sigma-Aldrich Japan, Tokyo, Japan), 1.5–8 mM Mg^2+^ (FUJIFILM Wako Pure Chemical Co., Tokyo, Japan), 300 µM dNTPs, 0.2 µM forward and reverse primers (Integrated DNA Technologies (IDT), Coralville, IA, USA) targeting a 76 bp product, 0.2 µM probe DNA (IDT), and 0.625 U GoTaq DNA polymerase (Promega K.K.). The primer set and HEX-labeled probe with BHQ-2 at the 5′ and 3′ ends, respectively, matched those employed in our prior studies [34,35].

### 2.2. Optimization of Mg^2+^ Concentration

The aforementioned PCR buffer, GoTaq Flexi Buffer, is specifically formulated as a Mg^2+^-free buffer, intended to facilitate the fine-tuning of Mg^2+^ concentration. This study involved a series of experiments aimed at optimizing the Mg^2+^ concentration in the PCR mixture. Initially, the Mg^2+^ concentration in the PCR reaction mixture was set at 1.5 mM, progressively increased to 2 mM, and then incrementally raised by 1 mM increments up to a final concentration of 8 mM. The response of droplet PCR was thoroughly investigated across this range of Mg^2+^ concentrations.

### 2.3. PDMS Microfluidic Sheet

**Figure 1:**
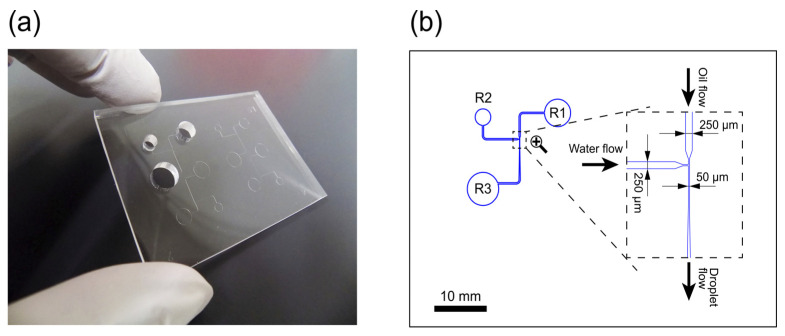
(**a**) Photo of a polydimethylsiloxane (PDMS) microfluidic sheet. (**b**) Drawing of a microfluidic channel pattern with the terminal reservoirs on the PDMS sheet and an enlarged view of the T-channel junction. R1 and R2 represent reservoirs for oil and aqueous phase loading, respectively, whereas R3 indicates an outlet space into which droplets generated at the T-channel junction flow.

Yodaka (Kanagawa, Japan) provided custom PDMS microfluidic sheets (70 mm × 50 mm × 4 mm) created through standard soft lithography techniques (Figure 1a). These sheets featured 50 µm deep channels with a T-junction, all made from PDMS. The T-junction design included a continuous phase (oil) channel and a dispersed phase (aqueous solution) channel intersecting at a right angle (Figure 1b). Along the microfluidic sheet, channel widths increased from 50 µm at the T-junction to 250 µm at the upstream and downstream points of the flow path. This design systematically diminishes the flow rate of produced droplets, preventing collisions between them. The channels extended to corresponding reservoirs (R1, R2, and R3 in Figure 1b). R1, R2, and R3 had capacities of 113, 50, and 201 µL, with diameters of 6, 4, and 8 mm, respectively.

### 2.4. Assembly of Microfluidic Droplet PCR Cartridge

**Figure 2:**
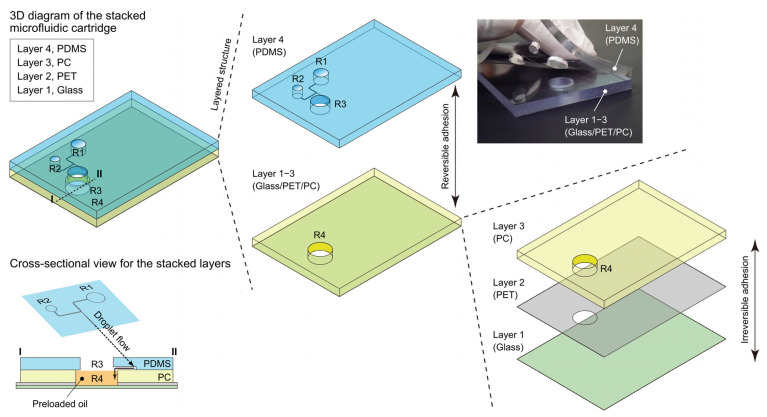
Illustration with a photographic image indicating the structure of the microfluidic cartridge, composed of four distinct layers: layer 1, thin glass (depicted in light green); layer 2, polyethylene terephthalate (PET) (depicted in light gray); layer 3, polycarbonate (PC) (depicted in light yellow); and layer 4, polydimethylsiloxane (PDMS) (depicted in light blue). Within layer 4, R1 and R2 denote reservoirs for oil and aqueous phase loading, respectively, whereas R3 represents an outlet space that is provided to establish a reduced-pressure environment by air suction for the fluid manipulation. The diagram at the lower left corner shows a cross-sectional view along (below) the dotted line I−II on the upper left 3D cartridge illustration.

The construction of the microfluidic cartridge comprised four distinct layers: a thin glass slide (Layer 1, situated at the bottom), a polyethylene terephthalate (PET) film coated with adhesive on both sides (Layer 2), a polycarbonate (PC) plate (Layer 3), and an uppermost layer consisting of a PDMS sheet (Layer 4) (See Figure 2). The PC plate (70 mm × 50 mm × 4 mm) featured a 10 mm diameter opening, serving as the PCR chamber (R4 in Figure 2), and it was affixed to a thin glass slide (70 mm × 50 mm × 0.15 mm) by means of a thin layer (0.1 mm thick) of double-sided adhesive PET film. This film possessed a punched hole with a diameter of 10 mm, strategically positioned just below R4. The aforementioned PDMS microfluidic sheet was attached to the PC plate through the reversible adherence facilitated by the viscoelastic properties of PDMS (consult the photograph in the upper right corner of Figure 2). In this configuration, R3 was situated directly above R4.

### 2.5. Procedures for Operation

**Figure 3:**
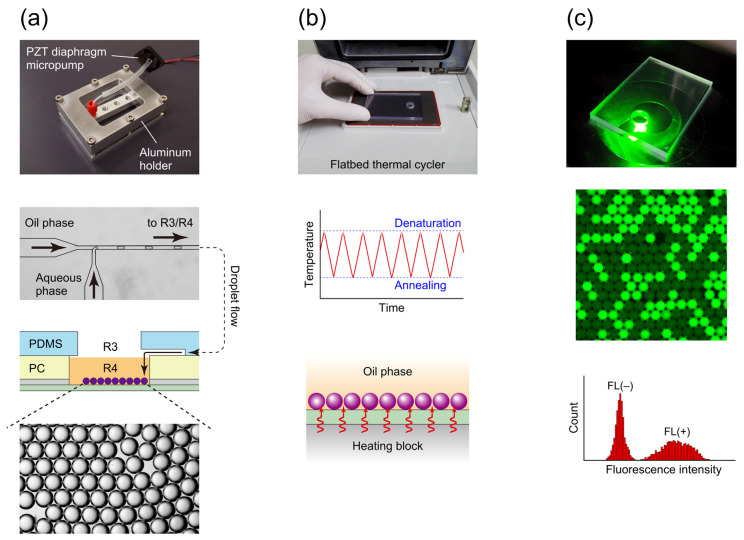
Schematic of the current droplet PCR workflow. (**a**) Droplet preparation, (**b**) PCR, and (**c**) fluorescence imaging of thermocycled droplets.

The operational steps in the current droplet PCR experiments are outlined schematically in Figure 3. Initially, the assembly of glass/PET/PC/PDMS underwent degassing in a vacuum desiccator at around 300 Pa for 15 min to facilitate fluid loading into the microchannels [25]. Upon returning the device to atmospheric pressure, 40 µL of an oil phase (mineral oil with 2% (*v*/*v*) ABIL EM 90 and 0.05% (*v*/*v*) Triton X-100) was dispensed into inlet reservoir R1, while 25 µL of the PCR mixture was added to inlet reservoir R2. Additionally, 320 µL of the oil phase was introduced into R4. Subsequently, the microfluidic device was integrated into the fluid manipulation system based on a piezoelectric (PZT) diaphragm micropump (see the top photographic image in Figure 3a) to induce fluid flow in the microchannels. Further information on the fluid control system can be found in our prior publications [34,36]. A drive voltage of 250 V_p-p_ at a frequency of 60 Hz was applied to the PZT micropump to evacuate air from outlet reservoir R3. The resulting pressure differential between the outlet and inlet reservoirs facilitated the movement of the preloaded oil (in R1, excluding R4) and aqueous solution into the channels, resulting in the formation of W/O droplets (67 µm in diameter) at the downstream T-channel junction (refer to the microscopic image near the center of Figure 3a). Due to the higher density of the discrete aqueous phase compared to the continuous oil phase (i.e., mineral oil), the W/O droplets descended from the channel terminus at the edge of R3 to the PCR chamber R4 of the PC layer. Subsequently, the aqueous droplets settled onto the glass slide positioned at the base of the chamber (see the lowermost microscopic image in Figure 3a).

Approximately 15 min after the initiation of droplet generation, the creation of droplets was halted, and the top PDMS layer was carefully lifted off the surface of the PC layer. Prior to removing the PDMS sheet, 160 µL of the oil phase was extracted from R4 to prevent spillage of the oil phase from R4. The resulting assembly of glass/PET/PC containing the reaction chamber was then transferred to a flatbed heating block of a thermal cycler (Mastercycler nexus flat, Eppendorf Japan, Tokyo, Japan) for 30 cycles of PCR amplification (refer to Figure 3b). Fluorescence images of the thermally cycled droplets were captured using an inverted fluorescence microscope equipped with an electron multiplier charge-coupled device camera (iXon^EM^+; Andor, Belfast, Northern Ireland) (see Figure 3c). Software bundled with the camera was employed to measure the fluorescence intensities of the droplets.

## 3. Results and Discussion

### 3.1. Temperature Response

The temperature of a sample within a microfuge tube in a heat block instrument reportedly lags tens of seconds behind the heat block temperature, depending on the sample volume in the tube (e.g., a time lag of 35 s for a 100 μL sample [37]). Therefore, in a representative commercial instrument, relatively long holding times of 30–60 s are usually required at denaturation, annealing, and extension temperatures for the sample to reach the block temperature. In line with this common approach, we provided ample holding times to the developed microfluidic cartridge in our previous research, such as 30 s at a denaturation temperature of 94 °C and 60 s at a combined annealing/extension temperature of 63 °C (hereafter termed standard cycling protocol) [35]. However, classical studies experimentally validated that denaturation and annealing can occur almost instantaneously (less than 1 s) once the sample reaches the appropriate temperature [28,37,38], which suggests that the denaturation and annealing segments of the temperature–time profile can be reduced to spikes (see the sketch-style drawing in the middle panel of Figure 3b, for instance) if quick temperature equilibrium of a PCR sample is warranted, leading to a substantial reduction in the time required for thermocycling. In the cartridge used in our previous study [35], small droplets (67 μm diameter; corresponding to 157 pL) with a high surface area-to-volume ratio of 89.6 mm^−1^ settled on a low thermal-capacity thin glass slide (recall that the thickness of the slide is only 0.15 mm) that directly contacts the heat block of the thermocycler, and momentary heat transfer from the block to the droplets can be expected, resulting in possible elimination of the temperature holding periods for denaturation and annealing. Primer extension is not instantaneous, but *Taq* DNA polymerase is highly processive, with an elongation rate of >60 nucleotides s^−1^ at 70 °C [39]. If a targeted region within a template sequence to be amplified is sufficiently short, as in the case of the present study (76 bp), complete elongation can be expected to occur during temperature transitions (i.e., annealing to denaturation temperature) [37].

Accordingly, we aimed to apply the following rapid thermocycling conditions to the droplets settled on the glass slide of the microfluidic cartridge: an ~40 s initial activation period at 94 °C to 98 °C, followed by 30 cycles of a two-step thermal profile involving <1 s at 94 °C for denaturation and <1 s at 63 °C for combined annealing and extension. These intended temperatures for denaturation (i.e., 94 °C) and annealing/extension (i.e., 63 °C) are identical to those in our previous study [35]. However, the dwell times at these temperatures are substantially briefer than those in the previous study (as aforementioned, 30 s at the denaturation temperature and 60 s at the annealing/extension temperature). The temperature on the glass slide is expected to change more slowly than on the metal heat block. To achieve temperature reversals without holding time at the target temperatures of 94 and 63 °C on the glass, we programmed a new thermal cycle for the block, with the holding time set to zero at temperatures exceeding those values. Specifically, the instrument was programmed to cycle between holding at 99 and 58 °C for 0 s each.

**Figure 4:**
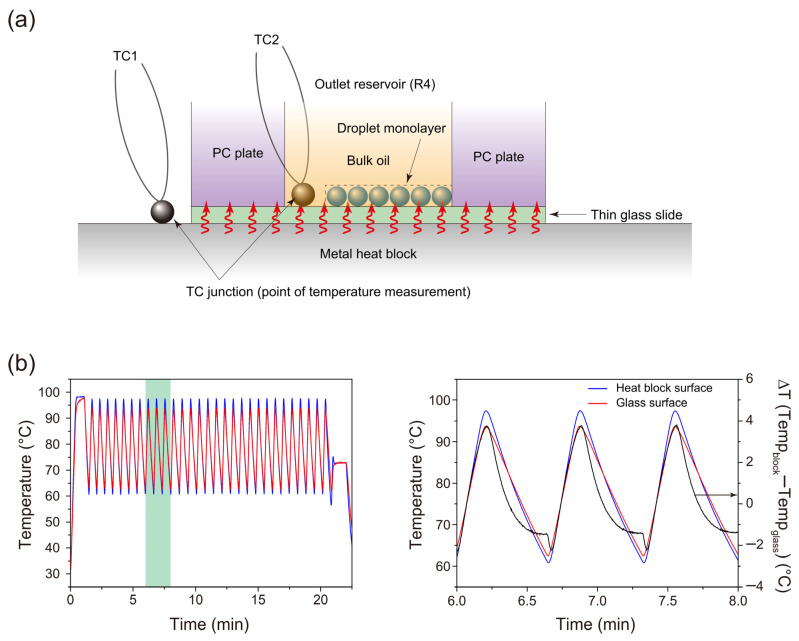
(**a**) Illustration of the PCR chamber (i.e., R4) with the thermocouples (TCs: TC1 and TC2) arranged to monitor temperatures on the metal heat block surface and the glass slide surface (with the covering oil), respectively, during thermal cycles. (**b**) Temperature vs. time traces for the surfaces of the aluminum heat block and glass slide. The thermal cycling conditions applied to the droplet samples were as follows: an initial activation period lasting approximately 40 s within the range of 94 to 98 °C, 30 cycles of a two-step thermal profile consisting of ~1 s at 94 °C for denaturation and ~2 s at 63 °C for combined annealing and extension, and an additional final extension of approximately 1 min at 72 to 73 °C. We obtained the profiles for the metal block surface (blue solid line) and for the glass slide surface (red solid line) upon programming the instrument to denature at 99 °C for 0 s and perform annealing/extension at 58 °C for 0 s at the fastest temperature ramp rate for the 30 cycles. The right panel exhibits an enlarged view of the green-shaded area shown in the left panel.

Simultaneously monitoring the actual temperatures of both the glass slide and the metal block, we carefully positioned two miniature thermocouple probes in direct contact with the surfaces of each of the materials to accurately reflect the temperature responses (refer to the illustration in Figure 4a). The temperature–time profiles of the metal block and the thin glass slide (with the covering oil) are depicted by the blue and red solid lines in Figure 4b, respectively. Through implementation of the temperature cycling program described previously (i.e., cycle programmed to hold at 99 and 58 °C for 0 s), we achieved thermal cycles characterized by brief dwell periods of ~1 s at 94 °C and ~2 s at 63 °C. These conditions closely aligned with our intended sequence. Rapid heat transfer, facilitated by the low heat capacitance of the thin glass slide, resulted in only slightly smaller temperature ramp rates on the upper surface of the glass slide (2.3 °C/s during heating and −1.2 °C/s during cooling) compared with those on the heat block surface (2.7 °C/s during heating and −1.4 °C/s during cooling). Implementation of this modified thermal cycle (hereafter termed fast cycling protocol), eliminating lengthy dwell times at the denaturation and annealing/extension temperatures, reduced the PCR amplification time from 67 min in our previous study [35] to 22 min. Despite this substantial time reduction, as elaborated in a subsequent section, we achieved PCR amplification from single-template DNA molecules within the droplets.

**Figure 5:**
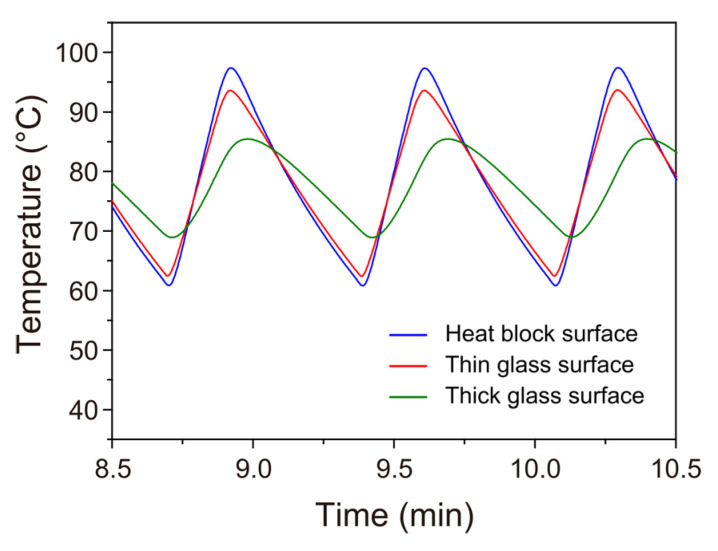
Comparison of temperature vs. time traces on the surface of the thick glass plate (1.1 mm thick, indicated by the green solid line) with those on the surfaces of the metal blocks (indicated by the blue solid line) and the thin glass slide (0.15 mm thick, indicated by the red solid line). We obtained the temperature profile for the thick glass plate surface upon programming the instrument to denature at 99 °C for 0 s, followed by annealing/extension at 58 °C for 0 s using the fastest temperature ramp rate, similarly to the measurements shown in Figure 4b.

Furthermore, Figure 5 shows the temperature–time profile resulting from the application of the same temperature program after replacing the bottom thin glass layer of the microfluidic cartridge with a glass plate of 1.1 mm thickness, alongside the temperature–time profile for the case of the thin glass slide as a comparative reference. The increase in glass plate thickness substantially lowered the temperature ramp rates (1.0 °C/s during heating and −0.7 °C/s during cooling), with the upper turnaround temperature reaching only 86 °C and the lower turnaround temperature reaching 69 °C, deviating considerably from the target temperatures of 94 and 63 °C, respectively. In the instance of utilizing the microfluidic cartridge featuring the thicker glass plate as the base, PCR amplification did not succeed.

### 3.2. Influence of Mg^2+^ Concentration in the Reaction Mixture on Droplet PCR Readout

In droplet PCR, at the end of the PCR amplification, each droplet is classified based on its fluorescence intensity into one of two groups: fluorescence-positive FL(+) droplets, which contain at least one target template molecule; and fluorescence-negative FL(−) droplets, which do not contain the target template molecule. By calculating the proportion of FL(+) droplets within the total number of analyzed droplets, the absolute copy number of the target DNA in the sample solution can be determined by Poisson regression. When presenting the results of the droplet PCR analysis as a histogram, with the vertical axis depicting the droplet count and the horizontal axis representing fluorescence intensity, distinct populations of FL(+) and FL(−) droplets become apparent in the histogram (see the bottom panel in Figure 3c, for instance). Accurate estimation of the numerical proportion of these FL(+) droplets relies on clear differentiation between these two populations. However, when the PCR amplification efficiency within the droplets is low, achieving a distinct separation of the FL(+) and FL(−) populations becomes challenging.

In PCR, magnesium ions (Mg^2+^) play a crucial role as cofactors in activating DNA polymerase. Generally, increasing the concentration of Mg^2+^ enhances the yield of PCR products [40]. However, an excessive Mg^2+^ concentration can lead to reduced specificity because of the stabilization of improper annealing of primers to incorrect template sites, potentially resulting in unintended PCR products [40,41]. Conversely, too low an Mg^2+^ concentration might lead to insufficient or no PCR product generation [40,41]. There is no single Mg^2+^ concentration that ensures PCR amplification in all situations, and the optimal Mg^2+^ concentration must be determined experimentally for each sample. Nevertheless, standard PCR protocols usually suggest a final Mg^2+^ concentration of 1.5 mM [42]. Many manufacturers’ DNA polymerases are supplied with 10× PCR buffer containing 15 mM Mg^2+^. Therefore, in our previous study [35], we performed droplet PCR by the standard cycling protocol starting with an Mg^2+^ concentration of 1.5 mM and also higher concentrations (up to 8 mM) to optimize the Mg^2+^ concentration. The fluorescence intensity of the FL(+) droplet population increased as the Mg^2+^ concentration increased from a value of 1.5 to a value of 2 mM and then to 3 mM, but thereafter, the fluorescence intensity of the FL(+) droplet population remained almost the same. In the present study, we investigated the effect of Mg^2+^ concentration on the separation of FL(+) and FL(−) droplet populations when applying the fast cycling protocol compared with that when applying the standard cycling protocol.

**Figure 6:**
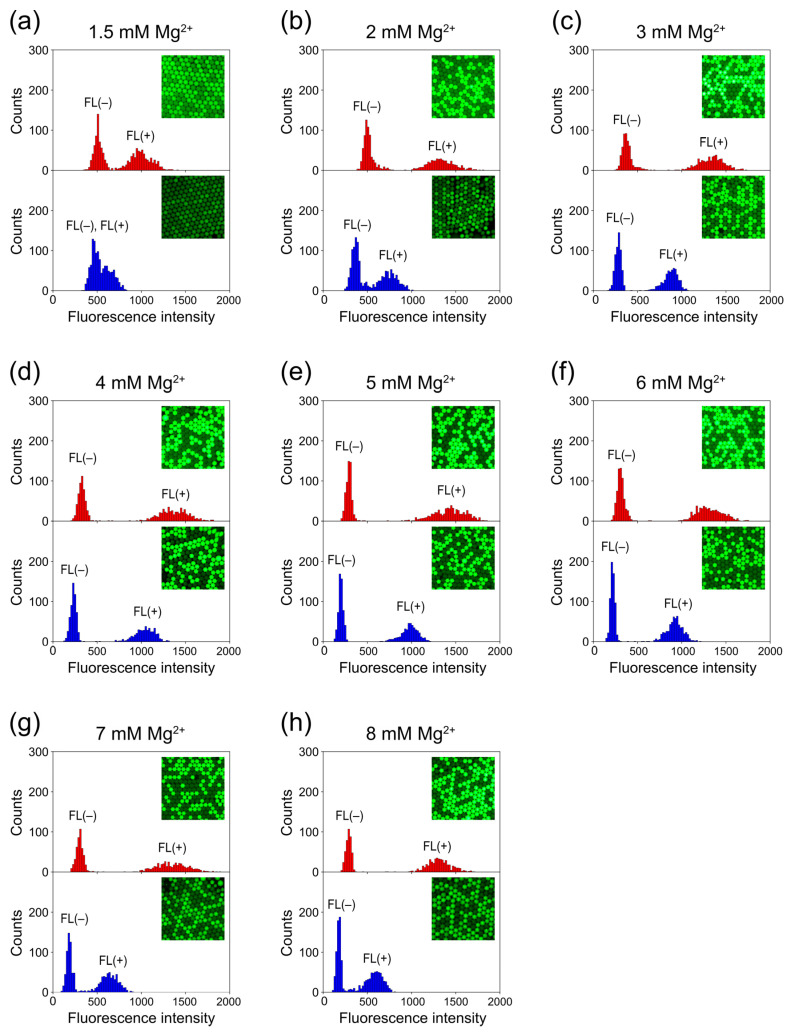
Influence of varying Mg^2+^ concentration on the degree of separation between FL(+) and FL(−) droplet populations in the standard and fast cycling protocols. We adjusted the ratio of the number of template DNA molecules to droplets to 7:10 for sample preparation. For each of (**a**–**h**), typical histograms obtained at each specified magnesium ion concentration ((**a**), 1.5 mM; (**b**), 2 mM; (**c**), 3 mM; (**d**), 4 mM; (**e**), 5 mM; (**f**), 6 mM; (**g**), 7 mM; and (**h**), 8 mM, respectively) are presented in the upper panel (depicted in red) for the standard cycling protocol and in the lower panel (depicted in blue) for the fast cycling protocol. The left cluster in each of the histograms indicates FL(−) droplets, and the right cluster indicates FL(+) droplets.

Consistent with our previous study [35], we systematically altered the Mg^2+^ concentration. We initiated with 1.5 mM, followed by 2 mM, incrementing each subsequent value by 1 mM until reaching 8 mM. Subsequently, we conducted droplet PCR experiments by the fast cycling protocol, repeating these trials multiple times. Figure 6 shows a comparison set of typical histograms obtained for each Mg^2+^ concentration under both protocols: upper panels for the standard protocol (performed in our previous study [35]) and lower panels for the fast protocol. At an Mg^2+^ concentration of 1.5 mM, the FL(+) droplet population was sufficiently separated from the FL(−) droplet population under the standard protocol, but not at all under the fast protocol (Figure 6a). At an Mg^2+^ concentration of 2 mM, the populations were completely separated under the standard protocol, but their distributions partially overlapped under the fast protocol (Figure 6b). At Mg^2+^ concentrations of ≥3 mM, even with the fast protocol, the FL(+) droplet population was completely separated from the FL(−) droplet population (Figure 6c–h). However, across all examined Mg^2+^ concentration conditions, the fast protocol consistently led to a higher distribution of FL(+) droplets in the lower fluorescence intensity region on the histogram, compared with the distribution observed when the standard cycling protocol was employed. This suggests a decrease in PCR amplification efficiency in droplets when we used the fast protocol. Although directly measuring the temperature inside the droplets would be ideal, it was technically challenging. As aforementioned, the temperature profile shown in Figure 4b (red solid line) reflects the temperature response of the oil phase, which covers the glass slide surface near the location where the droplets are placed (refer to Figure 4a for the location of the measurement point of the thermocouple (TC2)). We anticipated that the temperature inside the droplets would change nearly simultaneously with the temperature changes in the surrounding oil phase at that specific location.

However, heat transfer between the oil phase and droplets is not likely to be instantaneous, and the temperature inside the droplets might not reach the upper and lower target temperatures during the thermal cycle, resulting in a decrease in amplification efficiency. Even with the reduced PCR amplification efficiency, however, adequate separation of FL(+) and FL(−) droplet populations (observed when the Mg^2+^ concentration was ≥3 mM) enables estimation of the numerical proportion of FL(+) droplets, enabling absolute quantification of target molecules based on Poisson regression, as verified in the following section.

**Figure 7:**
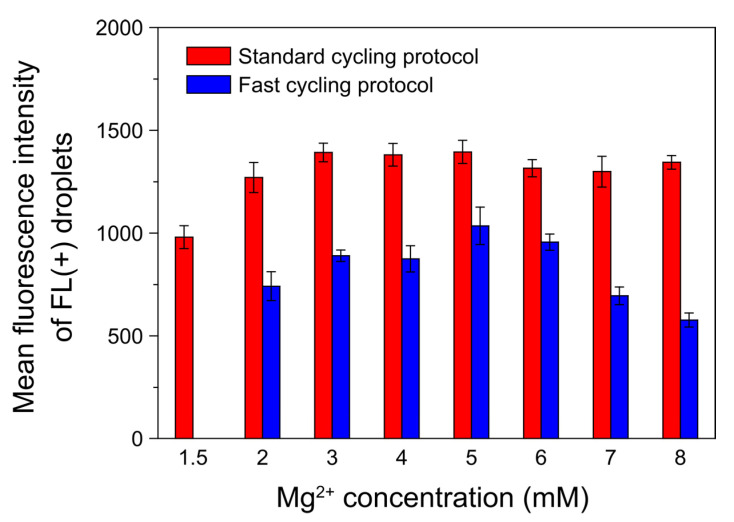
Influence of varying Mg^2+^ concentration on the mean fluorescence intensities of FL(+) droplets in the standard and fast cycling protocols. We adjusted the ratio of the number of template DNA molecules to droplets to 7:10 for sample preparation. The error bars represent SEM values. Number of experiments: *n* = 5.

Figure 7 summarizes the mean fluorescence intensities of FL(+) droplets resulting from five repeated experiments, each applying a standard cycle and a fast cycle at each Mg^2+^ concentration. Notably, under the application of the fast cycle, the fluorescence intensity exhibited a decline as Mg^2+^ concentrations exceeded 6 mM. This occurrence stands in contrast to the situation observed during the use of the standard cycling protocol. An excess of Mg^2+^ stabilizes DNA duplexes, preventing complete denaturation and reducing yield [40,41]. From this perspective, we speculate that the abbreviated duration spent at the denaturation temperature during the fast cycle hindered adequate denaturation of stabilized DNA duplexes because of the presence of surplus Mg^2+^, leading to this decrease in yield.

### 3.3. Droplet PCR from Single Molecules

As aforementioned, in droplet PCR, droplets containing one or more target template molecules fluoresce after PCR amplification and are classified as FL(+) droplets. The absolute quantification without standard curves is achieved by using the proportion of these FL(+) droplets based on Poisson statistics. In other words, the principle of absolute quantification remains valid only if PCR amplification succeeds, even when there is only a single template molecule in a droplet. As explained in Section 3.2, our experimental results demonstrate a potential reduction in PCR amplification efficiency when using the fast cycling protocol compared with the findings when using the standard cycling protocol. We conducted subsequent experiments to verify the viability of PCR amplification for a single molecule within a droplet, even under the conditions of reduced amplification efficiency resulting from the implementation of the fast cycling protocol. In consideration of the outcomes presented in Figure 7, we fixed the Mg^2+^ concentration at 5 mM for all sample preparations in the following experiments.

**Figure 8:**
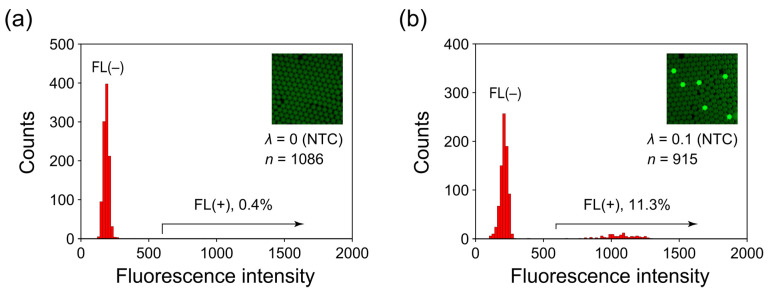
Histograms of hexachloro-fluorescein (HEX) fluorescence of thermocycled droplets for (**a**) no-template control (NTC) and (**b**) positive control experiments. The preset *λ* values, total droplet sample number, and percentage of fluorescence-positive FL(+) droplets in the distribution are indicated in the individual panels.

First, we prepared a no-template control (NTC) sample and randomly selected approximately 1000 droplets after PCR. We measured their fluorescence intensities and plotted a histogram (Figure 8a). Among all of the analyzed droplets, the proportion of FL(+) droplets was low: 0.4%. These observed false-positive droplets could be attributable to minor contamination of template molecules and/or infrequent formation of primer–dimer artifacts. We repeated this NTC experiment 5× separately, and in each instance, the measured proportion of FL(+) droplets remained <0.4%.

We then performed a positive control experiment by using a low copy number of template DNA molecules by the same procedure as the NTC experiment described previously. In accordance with the Poisson distribution, the distribution of the number of template DNA molecules that are encapsulated within droplets can be expressed with the following equation [14].

(1) $$p_{k}=\frac{{\lambda^{k}e}^{-\lambda }}{k!}.$$

Accordingly, the probability *p_k_* of encapsulating *k* DNA molecules in one droplet depends on the average number of DNA molecules per droplet (*λ*). We prepared samples such that the ratio of the number of template DNA molecules to the number of droplets was 1:10 (i.e., *λ* = 0.1). At this particular *λ* value, the majority of droplets (90.5%) contained no template molecules, whereas a small proportion of droplets (9.0%) contained only one template molecule. The proportion of droplets containing two or more template molecules was <0.5%. Consequently, most of the droplets that fluoresce correspond to those containing PCR amplicons derived from a single template molecule. Figure 8b shows an example of the fluorescence image obtained from the positive control experiment. A few droplets exhibited markedly stronger fluorescence compared with the others. Based on the aforementioned statistical inference, these highly fluorescent droplets corresponded to instances of PCR amplification stemming from a single template molecule. To verify whether the proportion of these highly fluorescent droplets concurred with the statistically inferred value at *λ* = 0.1, we constructed a histogram by using approximately 1000 randomly selected droplets (refer to Figure 8b). The proportion was 11.3%, within the 95% confidence interval (7.6% to 11.4%) predicted by the Poisson statistics for this particular *λ* value. Conducting the same experiment an additional 4× yielded proportions of fluorescent droplets measured at 9.4%, 10.1%, 9.7%, and 11.3%, all falling within the 95% confidence interval. The good agreement between the measured and Poisson-estimated proportions at this constrained *λ* value demonstrates that PCR amplification from single DNA molecules within droplets is attainable, even when applying the fast cycling protocol to our microfluidic cartridge.

## 4. Conclusions

The PCR amplification through thermocycling stands out as the most time-consuming step among the three major stages in the droplet PCR workflow. Despite this, as briefly mentioned in the Section 1, there have been very few studies attempting to expedite microfluidic droplet PCR tests by applying fast thermocycling [31,32]. Jalili et al. developed a plasmonic photothermal cycler and applied it to a microfluidic chip with a gold nanofilm, achieving rapid thermocycling of droplets within the chip reaction chamber [31]. The heating and cooling rates during the thermocycling were 7.4 °C/s and 1.9 °C/s, respectively, completing 30 thermal cycles between 60 °C and 90 °C within 13 min. Yin et al. also independently developed a sophisticated in situ heater array, completing a reverse transcription PCR consisting of 40 cycles in less than 5 min (7 s/cycle) [32]. In our current study, the PCR amplification process consisting of 30 thermal cycles took 22 min (40 s/cycle). Consequently, our thermocycling is slower than the thermocycling achieved in the studies of the two aforementioned groups. However, it seems that the fabrication of high-speed thermocycling systems used by these groups requires not only ample financial resources but also advanced technical expertise. In particular, developing the system by Yin et al. appears to necessitate an elaborate lithographic technique consisting of many complex steps with much equipment. In addition, the microfluidic chips used with those systems would need to be specially fabricated to work successfully with those systems, inevitably leading to high costs. Also, the PCR chamber of the chips developed by both groups is closed, making it difficult to extract the targeted droplets after PCR for use in downstream applications.

On the other hand, the microfluidic cartridge we developed can easily perform all three major steps in the droplet PCR workflow using common laboratory equipment, as highlighted in our previous publication [35], making it significantly more versatile than the microfluidic droplet PCR systems of the above two groups. As demonstrated in this study, even with the application of the fast thermocycling protocol, PCR amplification from single molecules was achieved, allowing for highly accurate quantification based on Poisson regression. The heating and cooling rates of commercially available thermal cyclers have progressively increased over the years. Reducing the cross-sectional dimensions of the microchannels results in smaller droplets. This decrease is expected to enhance the heat transfer rate from a heating block of a thermal cycler due to the increased surface area-to-volume ratio of the droplet. Consequently, the droplet temperature is likely to closely track the rapid heating and cooling rates of an upcoming high-speed thermal cycler. Hence, we anticipate achieving even faster single-molecule droplet PCR without compromising PCR efficiency in the near future using our microfluidic cartridge. Furthermore, our PCR chamber, made by simply adhering an inexpensive PC plate to thin glass with double-sided tape, not only poses no cost challenges for a one-time use but also allows for easy and selective collection of droplets after PCR from the open-top reaction chamber. This makes it possible to perform downstream applications (e.g., aptamer selection [18]). Additionally, our microfluidic sheet, reversibly attached to the PCR chamber using the viscoelastic properties of PDMS, can be reused with simple rinsing between runs.

## Data Availability

Data are contained within the article.
